# Survivorship from pediatric and adult brain tumors: The 2024 Brain Tumor Epidemiology Consortium meeting report

**DOI:** 10.1093/noajnl/vdaf095

**Published:** 2025-05-20

**Authors:** Scott L Coven, Roberta McKean-Cowdin, Michael E Scheurer, Kimberly J Johnson, Luc Bauchet, Carol Kruchko, Ching C Lau, Quinn T Ostrom, John Villano, Yan Yuan, Friederike Erdmann

**Affiliations:** Divison of Pediatric Hematology/Oncology/Stem Cell Transplant, Department of Pediatrics, Indiana University School of Medicine and Riley Children’s Health, Indianapolis, Indiana, USA; University of Southern California, Los Angeles, California, USA; Department of Pediatrics, Division of Hematology/Oncology, Baylor College of Medicine, Texas Children’s Hospital, Cancer and Hematology Centers, Houston, Texas, USA; Brown School Master of Public Health Program, Washington University, St. Louis, Missouri, USA; Montpellier University Hospital, Montpellier, France; Central Brain Tumor Registry of the United States, Hinsdale, Illinois, USA; Connecticut Children’s Medical Center, Hartford, Connecticut, USA; The Jackson Laboratory for Genomic Medicine, Farmington, Connecticut, USA; Department of Neurosurgery, Duke Cancer Institute, and The Preston Robert Tisch Brain Tumor Center, Duke University School of Medicine, Durham, North Carolina, USA; University of Kentucky School of Medicine, Lexington, Kentucky, USA; University of Alberta, School of Public Health & Women and Children’s Health Research Institute, Edmonton, Alberta, Canada; Department of Prevention and Evaluation, Leibniz Institute for Prevention Research and Epidemiology – BIPS, Bremen, Germany; Research Group Aetiology and Inequalities in Childhood Cancer, Division of Childhood Cancer Epidemiology, Institute of Medical Biostatistics, Epidemiology and Informatics (IMBEI), University Medical Center of the Johannes Gutenberg University, Mainz, Germany

**Keywords:** brain tumors, epidemiology, survival, survivorship

## Abstract

The Brain Tumor Epidemiology Consortium (BTEC) is an international organization with membership of individuals from the scientific community with interests related to brain tumor epidemiology, including surveillance, classification, methodology, etiology, and factors associated with morbidity and survival. The 2024 annual BTEC meeting entitled “*Survivorship from Pediatric and Adult Brain Tumors*” was held in Mainz, Germany, USA, on May 15–17, 2024. The meeting gathered scientists from Africa, Australia, Europe, and North America and included 4 keynote sessions focusing on brain tumor survivorship across the age spectrum. The meeting included 3 abstract sessions, which also included scientific talks around brain tumor risk factors and predicting risk and survival. We also held a brainstorming session to form a near-term research strategy around brain tumor survivorship in the epidemiology community. This report provides a summary of the meeting content.

The Brain Tumor Epidemiology Consortium (BTEC) was founded in 2003 following a meeting sponsored by the U.S. National Cancer Institute’s (NCI) Division of Cancer Epidemiology and Genetics and the U.S. National Institutes of Health’s (NIH) Office of Rare Diseases. In 2013, BTEC was incorporated as a nonprofit with 501(c)(3) status. The 2024 annual meeting was held in Mainz, Germany, on May 15–17 and was titled “***Survivorship from Pediatric and Adult Brain Tumors***.” *The program committee included Board of Director members: co-Presidents Michael Scheurer, PhD, MPH and Yan Yuan, PhD; co-Vice-Presidents Jon Foss-Skiftesvik, MD and Roberta McKean-Cowdin, PhD, MPH; Treasurer Quinn Ostrom, PhD, MPH; and Secretary Carol Kruchko. Presentations included eight keynote addresses and 22 oral abstract presentations including two American Brain Tumor Association (ABTA) Junior Investigator Award presentations (US and non-US). Ms. B*énédicte Clement of Montpellier, France, was the meeting coordinator. In this report, we briefly summarize the meeting content. Abstracts can be found at https://sites.usc.edu/braintumorcause/program-with-abstracts-btec-mainz-2024/.

## Day 1

Day one of the conference began with welcoming remarks by the BTEC Co-Presidents Michael Scheurer, PhD, MPH of Baylor College of Medicine (Houston, TX, USA) and Yan Yuan, PhD of the University of Alberta (Edmonton, AB, Canada) and conference host Friederike Erdman, PhD, MPH of the Institute of Medical Biostatistics, Epidemiology and Informatics (IMBEI), University Medical Center Mainz (Mainz, Germany). The session opened with 3 keynote lectures on trends in brain tumor survival and survivorship and health issues for survivors of adult and pediatric brain tumors.

Fabio Girardi, PhD, of the London School of Hygiene and Tropical Medicine (London, UK) began the session with a keynote lecture on ***“Survival trends for adults and children diagnosed with brain tumors*.”** Dr. Girardi noted that population-based survival is a key metric to evaluate the effectiveness of a healthcare system in managing all patients diagnosed with cancer. The presentation centered around data from CONCORD-3, a program established for global surveillance of cancer survival, which includes individual records for 37.5 million cancer patients from 2000 to 2014.^1^ Dr. Girardi described that disparities in survival observed by country are influenced by a range of factors, including symptom recognition, diagnosis, treatment, follow-up care, and palliative care. Using CONCORD-3 data, age-standardized 5-year net survival in children varied widely between countries during 2000–2014, ranging from 80% to 100% for low-grade astrocytoma and 6%–30% for high-grade tumors.^2^ In adults, trends in 5-year net survival were relatively stable during 2000–2014; however, some improvements were observed in short-term 2-year net survival for glioblastoma.^3^ Dr. Girardi also emphasized that accurate monitoring of cancer outcomes requires histologic-based survival estimates, but acknowledged the challenge given the global variation in cancer registration. Large variation in rates of histologic subtypes of childhood brain tumors (CBT) also have been reported by country.^4^ Survival rates in countries that only include malignant brain tumor diagnoses are systematically lower than in countries that include malignant and nonmalignant cases. Dr. Girardi noted survival reporting by the Central Brain Tumor Registry of the United States (CBTRUS) is histology based, but many countries do not have the resources to complete this level of reporting.^5^ CBTRUS has begun to report incidence and survival by molecular markers, which likely represents the future standard for survival evaluation. In conclusion, Dr. Girardi stated that the usefulness of registry data when monitoring brain tumor survival will depend on the completeness and accuracy of the data. There is a need for high-quality cancer data from low- and middle-income countries, and there are currently disparities in both access to high-quality health care, cancer registration practices, and the accuracy of the pathological diagnoses. A cohesive reporting on a broad scale will bring impactful change to patients with central nervous system (CNS) tumors around the globe.

Keynote lecture two was presented by Paul Nathan, MD, MSc of the University of Toronto Sick Kids (Toronto, Canada) on ***“Current topics in late effects of childhood brain tumor therapy*.”** He described the paradigm shift away from “cure at any cost” to “quality of the cure.” As more children survive after a brain cancer diagnosis, the impact of treatment on kids who survive grows in importance. Late effects and adverse consequences in later life are numerous, including mortality, subsequent malignant neoplasms, chronic health conditions, neurocognitive deficits, mental health problems, adverse psychosocial outcomes (eg, education, employment, marriage), functional limitations, and financial hardship. He discussed that the Childhood Cancer Survivor Study found lower mortality but higher morbidity for survivors of medulloblastoma and astrocytoma over time from the 1970s to 1990s.^6^ Dr. Nathan also explained that risk of cognitive impairment and neuropsychological deficits for brain tumor survivors is influenced by tumor-related, treatment-related, and patient-related factors as well as environmental factors. He challenged the audience to consider how we offer care to survivors in our communities, given that most primary care physicians do not have experience caring for pediatric brain cancer survivors. He noted that the likelihood of patients continuing visits to specialists at their cancer center drops substantially over time, while the risk of late effects continues to increase over time. He discussed that interventions to provide good care for survivors could include primary prevention such as efforts to minimize and limit exposure to radiation. Guidelines for secondary prevention for surveillance and optimization of quality of life (QOL) should follow international guidelines including those established by the Children’s Oncology Group or the International Guideline Harmonization Group. Tertiary preventions may include pharmacologic, exercise, or behavioral interventions, but have been less successful in substantially changing the long-term effects of childhood brain tumor therapy.

The third keynote lecture was given by Dr. Florien Boele of the Leeds Institute of Health Sciences (Leeds, UK) on health issues of adult brain tumor survivors. Dr. Boele spoke about health-related quality of life (HRQOL) for adult survivors of brain cancer and that survivorship becomes more meaningful to patients if there is preservation of function and well-being. She noted that the burden of symptoms is frequent and prevalent, including seizures, cognitive deficits, drowsiness, dysphagia, headache, confusion, aphasia, motor deficits, and fatigue. The most severe symptoms include fatigue, disturbed sleep, distress, sadness, pain, memory issues, irritability, and difficulty speaking. She compared the types of changes in HRQOL by type of brain tumor (meningioma, low-grade glioma, and high-grade glioma) and by stage of treatment (treatment, early stage, long term). She noted there are increasing HRQOL impacts with increasing severity of disease across domains; however, treatment can have positive or negative impacts on HRQOL. For example, resection may decrease immediate symptoms due to the removal of tumor tissue and improve HRQOL or it may introduce new deficits. Similarly, chemotherapy may introduce worse short-term deficits and better long-term effects. Overall, symptom burden during treatment is high and many domains of HRQOL are impacted. During follow-up, HRQOL can return to the same level as healthy controls, but severe limitations in physical, mental, emotional, or social domains can persist. Fatigue is a frequent and severe symptom that can be debilitating to survivors. Support of psychological adaptation can be important to avoid feelings of loss of autonomy and independence, where patients focus on positive outcomes and a shift in priorities. To assist brain tumor patients, future efforts should be aimed at complex psychosocial interventions. Dr. Boele concluded that “there is much that we can’t change for patients, but we can improve the symptom burden and prevent psychological maladjustment.” She urged that we seek improvements in predicting who is at risk for worsening HRQOL and become better at implementing tailored, complex, multidisciplinary interventions to optimize patient QOL.

Mid-morning and mid-afternoon abstract presentation sessions focused on the pediatric and adult brain tumor research of the attending members. Speakers and presentation topics for Day 1 are shown in Table 1.

**Table 1.** Abstracts presented at the 2024 BTEC conference

Presenter	Institution	Title
**Day 1**		
Maike Wellbrock	Institute of Medical Biostatistics, Epidemiology, and Informatics—Germany	Survival Patterns of Pediatric Central Nervous System Tumors in Germany 1980-2016: A Nationwide Assessment Based on Data from the German Childhood Cancer Registry
Luc Bauchet, MD, PhD	CHU Montpellier—France	Epidemiology, Clinical Data, and Long-Term Functional Outcomes for Operated Central Neurocytoma Patients: Preliminary Results of the French Experience
Louise Tram Henriksen	Aarhus University Hospital—Denmark	Standard of Care for Follow-Up for Neurovascular Late Effects After Radiotherapy for Pediatric Brain Tumors
Sarah Al Sharie	Yarmouk University—Jordan	The Epidemiology of CNS Tumors in Arab Countries: A Globocan Database Analysis
Angela Lopez-Cortes	University College London—UK	International Benchmarking of Childhood Cancer Survival by Stage (BENCHISTA Project): Results for Medulloblastoma
Syed A. Sarwar	Jersey Shore University Medical Center—USA	Temporal Variation in Glioblastoma Multiforme Across the United States: Incidence Increases from 2000 to 2017
Julia Botvinov	Hackensack Meridian School of Medicine—USA	Impact of Maternal Health and Demographics on Childhood Brain Tumor Incidence: An Epidemiological Study
**Day 2**		
Joshua D. Strauss ABTA Junior Investigator Award Recipient (US)	Baylor College of Medicine—USA	Novel Susceptibility Variants in Adult and Pediatric Ependymoma
Raoull Hoogendijk ABTA Junior Investigator Award Recipient (non-US)	Princess Máxima Center for Pediatric Oncology – Netherlands	Geographical Survival Comparison and Estimated Long-Term Survival Outcomes of Pediatric CNS Tumors from 31 European Countries—Results from the Population-Based EUROCARE Project
Emily Nissen	University of Kansas Medical Center—USA	Enrichment of a Neutrophil-Like Monocyte Transcriptional State in Glioblastoma Myeloid Suppressor Cells
Scott L. Coven	Riley Hospital for Children at IU Health—USA	Establishing a “Medical Home” for Children with Incidental Brain Lesions
Thanh T. Hoang	Baylor College of Medicine—USA	Association Between Congenital Anomalies and Childhood Brain Tumors in 22 Million Live Births
Mackenzie Price	Central Brain Tumor Registry of the United States—USA	Multiscale Geographically Weighted Linear Regression for County-Level Glioblastoma Incidence Modeling in the United States
Yan Yuan	University of Alberta—Canada	Investigating Diagnostic Cost Disparities for Central Nervous System Tumours Across Canadian Provinces
Karen Alpen	University of Melbourne—Australia	Investigating the Role of Germline DNA in the Brain Location of Adult Glioma Tumors
Christina-Evmorfia Kampitsi	Kaolinska Institutet—Sweden	Parental Occupational Exposures and De Novo Neurocutaneous Syndromes in the Offspring: Findings from a Register-Based Swedish Case-Control Study
Bin Huang	University of Kentucky—USA	Characteristics of Mental Health Disorder Among Central Nervous System Cancer and Other Childhood Cancer Patients—A Population-Based Study for Medicaid Beneficiaries in Kentucky
Aizpea Artetxe Zurutuza	Biogipuzkoa Health Research Institute—Spain	Characterization of Novel Multi-Target Compounds for the Treatment of Glioblastoma
Victoria Mwebe Katasi	Mulago National Referral Hospital—Uganda	Descriptive Epidemiology of Childhood Primary Brain Tumors in Uganda: Data from a Tertiary Center
Javier Louro	Karolinska Instiutet—Sweden	Long-Term Antidepressant Drugs Use Among Childhood CNS Tumor Survivors in Sweden: A Register-Based Cohort Study
Christine Ann Pittman Ballard	University of North Carolina—USA	The Effect of Prior Varicella Zoster Infection on a Person’s Risk of Glioma Development
Stephanie Chen	UCLA—USA	Maternal Exposure to Solvents from Industrial Sources During Pregnancy and Childhood Cancer Risk in California
Mantas Dmukauskas	National Cancer Institute—USA	Sex Differences in Adverse Events Post Treatment in Glioblastoma
Sara Hadad	University of California San Francisco—USA	De Novo Replication Repair Deficient Glioblastoma, IDH-Wildtype is a Distinct Glioblastoma Subtype in Adults That May Benefit from Immune Checkpoint Blockade

The keynote lectures continued, after the presentation of morning abstracts, with Dr. Andrea Pace, MD from the IRCC Regina Elena National Cancer Institute (Rome, Italy) on ***“Psychosocial and mental health outcomes among cancer survivors*.”** Dr. Pace reported that many brain tumor survivors suffer from cognitive deficits, which in turn further compromises their HRQOL. A key challenge in assessment of long-term effects and QOL in brain tumor survivors is the lack of standardized, validated tools for use across studies. The CCSS Neuro-cognitive questionnaire (CCSS-NC) was developed to evaluate neurocognitive status in adult long-term survivors of childhood CNS malignancies in the Childhood Cancer Survivor Study.^7^ The CCSS-NC assesses several key neurocognitive domains: task efficiency, emotional regulation, organizational skills, and memory. Among adult survivors of CNS tumors, Dr. Pace reported that subjective cognitive function was significantly lower than controls, and patients reported greater anxiety, fatigue, and treatment-related side effects. Factors found to predict neurocognitive deterioration included younger age at diagnosis, sex, radiotherapy, chemotherapy, ventricular shunt, and neurological complications during therapy. In particular, brain irradiation is thought to impact long-term cognitive disability by direct mitochondrial DNA damage or secondarily through the production of reactive oxygen species and free radicals. Because of the impact that neurocognitive effects have on overall QOL, investigators are evaluating neuroprotective strategies to overcome these effects, including symptomatic treatments, putative neuroprotective strategies, and cognitive rehabilitation. Dr. Pace concluded that cognitive function assessment and rehabilitation strategies are fundamental in this population of patients. However, more data are needed to assess the incidence of supportive care issues such as mood disorders, infertility, marriage status, and psychosocial needs in long-term brain tumor survivors.

The last keynote lecture for Thursday morning was given by Kiri Ness, PhD, of the St. Jude Children’s Research Hospital (Memphis, TN, USA) titled **“*Dealing with disability as a brain tumor survivor*.”** Dr. Ness noted that hand grip strength (kg) and peak oxygen uptake (ml/kg/min) in adult survivors of a CBT, as 2 of the strongest predictors of mortality in this general population. She also noted that ability to complete activities of daily living is worse among brain tumor survivors than population-based controls. The provision of underlying support to maximize cardiopulmonary fitness is important so that CBT survivors are better able to cope with chronic neurological impairments. Dr. Ness found that measures of brain activation are correlated with higher peak oxygen intake (PKVO2), indicating that the brain functions better with better fitness. Further, voluntary physical exercise has beneficial effects on memory and cognition. To explore the potential benefits of physical exercise, Dr. Ness conducted a randomized pilot intervention for 12 weeks on children undergoing treatment for medulloblastoma, where children either received the intervention and physical therapy or physical therapy only. The intervention included 30 minutes of exercise within the target heart rate zone for 3 days a week over 10–12 weeks. The children achieved a mean improvement in PKVO2 for aerobic training and physical therapy compared to physical therapy only (+2.11); however, the difference did not achieve statistical significance due to the small sample size of the pilot study. She also found that higher PKVO2 was associated with better cognition. Dr. Ness also noted that participants with higher PKVO2 had faster reaction times and that cerebral blood volume increased after exercise. The findings suggest that exercise is safe and effective during therapy for children with medulloblastoma.

The morning session was followed by a lunch meeting for all attendees and a discussion of the BTEC Advisory Board Meeting. The afternoon session began with abstract presentations and discussions, chaired by Quinn Ostrom, PhD (Duke University, Durham, NC, US) and Ching Lau, MD, PhD (The Jackson Laboratory, Framingham, CT, US) on survivorship and epidemiology of adult and pediatric brain cancer.

The afternoon keynote sessions opened with a presentation by Dr. Vidya Puthenpura from Yale University (New Haven, Connecticut, US) on the **“*Social determinants of brain tumor survivorship in children*.”** Dr. Puthenpura spoke about achieving health equity for survivors of CBT and the role of several sources of survivorship inequities, including race and ethnicity, economics, geography, sexual orientation, and gender identity. With respect to race and ethnicity, Dr. Puthenpura used Surveillance, Epidemiology and End Results (SEER) data to evaluate survival disparities, finding longer 5-year survival for non-Hispanic white children relative to children from other racial and ethnic groups.^8^ She noted that the large number of histologic types (>100) of pediatric brain tumors made exploring the causes of health disparities complex. Dr. Puthenpura spoke about contributing factors including limited data on survivorship by race and limited published data on structural racism and psychosocial barriers to cancer care for pediatric brain tumor cases. She discussed that low economic resources, particularly in the United States, are associated with worse health outcomes and often include the presentation for care at later stages of cancer, not receiving preventive medical care, lower adherence to therapy, decreased access to clinical trials, worse access to adequate nutritious food, challenges in transportation, and the need for supportive housing care.^8,9^ In the second half of the presentation, Dr. Puthenpura transitioned her talk to strategies for addressing health inequities in neuro-oncology survivorship care. She highlighted that clinicians and researchers need to build enduring and trusting relationships with patients and community partners and that interventions need to be developed with inclusivity of diverse populations. Health-related social needs data should be collected as part of collaborative working groups such as the Children’s Oncology Group (COG), comprehensive tumor registries such as the Molecular Characterization Initiative, and pediatric clinical trials, such as the Pediatric Neuro-Oncology Consortium. Digital health care may serve to decrease health inequities in the future by improving access to diagnostic care, enhancing treatment options, and improving data collection on social determinants of health.^10–12^ Digital health care, for example, may improve access to physical therapy at a patient’s home and provide access to specialized care and tailored rehabilitation programs, with several pilot programs under investigation in the United States.^13^ Virtual reality support groups for adolescents and young adults have been tested and provide feedback and improve resiliency, anxiety, depression, and positive affect.^14^ Improving inequities in pediatric cancer survivorship will require a holistic approach that includes not only medical treatment, but also psychosocial, educational, and economic support. Efforts are needed to foster partnerships between healthcare providers, community organizations, and technology innovators to develop accessible and effective solutions.

The final keynote lecture of day 1, was given by Nicole Willmarth, PhD, of the ABTA (Chicago, IL, US) on ***“Financial toxicity among brain tumor survivors*.”** Dr. Willmarth spoke based on information gathered by the ABTA, the oldest patient advocacy agency in the United States for brain tumor patients, to educate patients and their families on how to manage financial burden of a brain tumor diagnosis. Financial toxicity refers to out-of-pocket expenses related to treatment that can diminish QOL and impede delivery of the highest quality of care. Dr. Willmarth described that both objective financial burden and subjective financial distress are key components of financial toxicity. She further described findings from the 2019 National Cancer Opinion Survey (United States) that finances caused anxiety in 7 of 10 cancer patients and caregivers and that 2 of 5 caregivers reported actions to reduce treatment costs, such as postponing filling prescriptions, skipping doctor appointments, and missing scans that ultimately impact patient care and outcomes.^15^ She noted that patients were often forced to make decisions between lifesaving treatments, inability to afford timely care, and potential bankruptcy. In fact, cancer patients were 2.6 times as likely to declare bankruptcy compared to those without a cancer diagnosis and those who declared bankruptcy were 80% more likely to die than cancer patients who did not.^16,17^ Dr. Willmarth explained that financial toxicity was associated with decreased patient satisfaction with care, QOL, and quality of care. Factors that influence financial toxicity include rising healthcare costs, increased cost sharing for patients, inadequate insurance coverage, and payment issues. She then spoke about how the ABTA is working to assist brain tumor patients to prevent financial toxicity in the first place. One goal is to improve patient’s understanding of the “language” of insurance to empower patients and caregivers to secure the most benefit from their insurance. For example, for patients in the United States, 14% of medical claims are denied and few are appealed, even though up to 90% of claim denials are wrong. Cancer patients can appeal denied payments and many times institutions can work with an insurance company if there is a denial to secure insurance approvals on behalf of patients.^18^ Another goal is to improve policy to maximize patient benefits. De-escalation research is needed to evaluate how we can do more for patient care with less. For example, testing for the lowest effective dose rather than the maximum tolerated dose. Comparative effectiveness research is needed to evaluate the effectiveness of less relative to the more expensive treatment options. Patients also need to work with financial navigators to help optimize insurance use and to have open conversations with clinical doctors on ability to pay for treatments.^19^ Dr. Willmarth concluded that patients should not be in the position of deciding between death or bankruptcy, or in the worst cases—both.^20^

## Day 2

Day 2 commenced with a session on new approaches to cancer investigation. Annette Molinaro, PhD, of UCSF (San Francisco, California, USA) gave a talk on **“*Using Machine Learning and AI to Predict Brain Cancer Survival.*”** Dr. Molinaro spoke about the opportunities to utilize artificial intelligence (AI) and machine learning (ML) in neuro-oncology (Khalighi), with examples including an integrated diagnosis, leveraging MRI images for automated detection, aiding surgical precision, analyzing fresh pathology samples, and streamlining the molecular profiles. Dr. Molinaro explained that limitations exist regarding AI and ML, owing to the complexity of algorithms being specific to the classification or the linear outcome that is observed. Additionally, her work has focused on survival outcomes, and this can often be a challenge when it is binarized to “dead” or “alive.” She then shared several of the project areas she has been involved with AI and ML. As introduced in the recent paper by Khalighi et al., Dr. Molinaro and her team have been working to predict survival for patients with isocitrate dehydrogenase (IDH) mutated gliomas with respect to extent of resection.^21,22^ Using AI and ML algorithms, they found a more extensive resection for low-grade glioma patients led to improved overall survival. She described some recent work for an immune profiles study for newly diagnosed glioblastoma multiforme, where they are using machine learning techniques on methylation arrays on the blood. Lastly, she shared a project to prognosticate glioma survival using complex neural networks for patients with gliomas based on digital pathology images.^23^

The second lecture titled ***“Brain Tumor Classification by Enzymatic DNA Methylation Sequencing of Cell Free DNA from Cerebrospinal Fluid”*** was given by Ching Lau, MD, PhD, from The Jackson Laboratory (Farmington, Connecticut, USA). Dr. Lau started by describing the current unmet needs in brain tumor care. He explained that a tissue diagnosis can be challenging for deep-seated lesions, such as diffuse midline gliomas, hypothalamic gliomas, optic pathway gliomas, and intracranial germ cell tumors, and that treatment sometimes needs to be initiated without a definitive diagnosis. Furthermore, disease response is often dependent on imaging alone. Dr. Lau was specifically interested in intracranial germ cell tumors, given that a tissue biopsy can be limited in giving one the “whole picture,” especially for patients with nongerminomatous germ cell tumors. His lab described novel somatic and germline mutations in c-KIT and KRAS with intracranial germ cell tumors.^24^ This work led to discussions on how to complement the tissue diagnosis with cerebrospinal fluid (CSF) or plasma/serum profiling, finding CSF as a better choice due to its contact to the tumor, lower contamination, and harboring more tumor-related DNA. They have been successful in demonstrating characterization between germinoma and nongerminomatous germ cell tumors through microRNA samples, although further subtyping of nongerminomatous germ cell tumors has been problematic. Dr. Lau’s lab has also used additional techniques, such as DNA methylation, demonstrating the ability to classify and subtype for germinoma samples. However, these techniques are proving more challenging for the nongerminomatous subtypes likely due to their complex and heterogenous histology. The utilization of liquid biopsy could provide opportunities for monitoring clinical response, monitoring disease progression, and evaluating “minimal residual disease,” such as in leukemia. Dr. Lau’s team has demonstrated the need for a liquid biopsy platform to complement the tissue diagnosis, and stated that an ideal platform would likely integrate multi-omic techniques (DNA methylation, copy number variation, miRNA, and mutational profiles).

**Figure 1.**
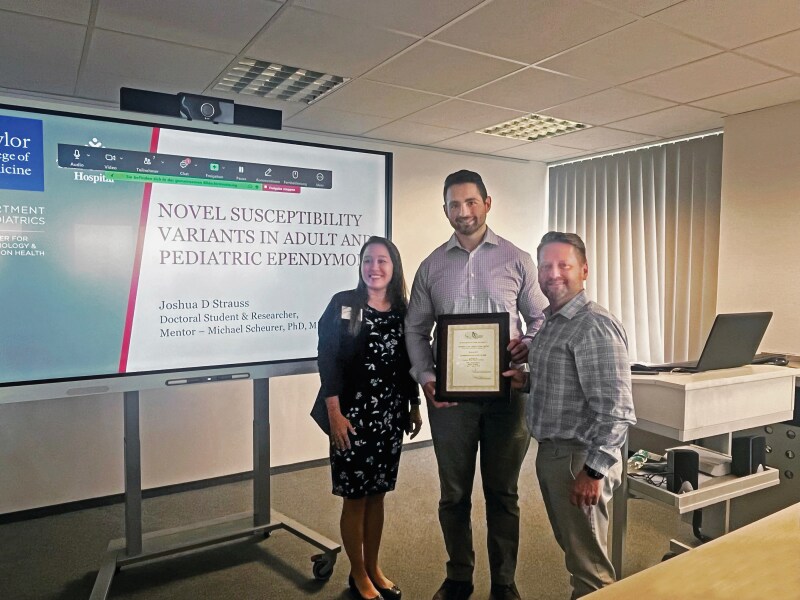
American Brain Tumor Association (ABTA) Junior Investigator Award presentations. Pictured from left are ABTA chief mission officer Nicole Willmarth, PhD, doctoral student and junior investigator winner Joshua D Strauss, and BTEC President Michael Scheurer, PhD. Bénédicte Clement took the photographs and approved permission for use.

**Figure 2.**
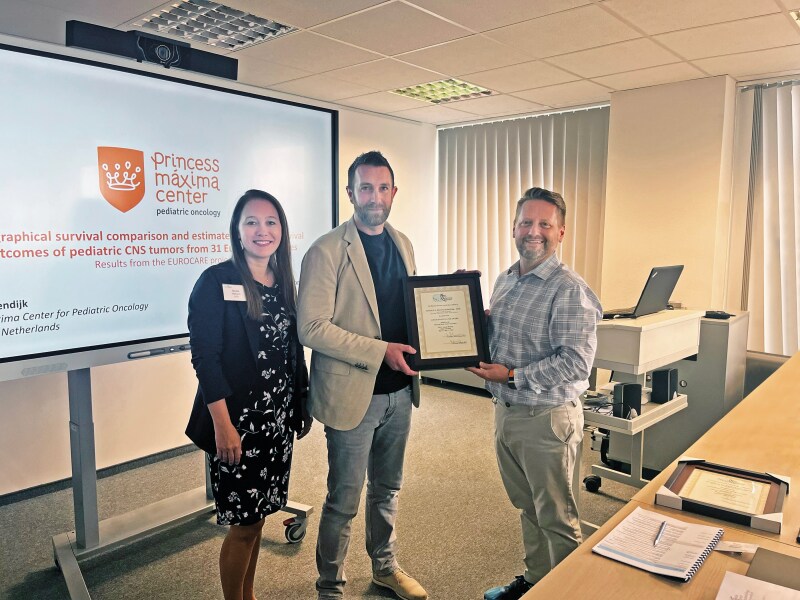
American Brain Tumor Association (ABTA) Non-US Junior Investigator Award presentations. Pictured from left are ABTA chief mission officer Nicole Willmarth, PhD, Non-US junior investigator winner Raoull Hoogendijk, PhD, and BTEC President Michael Scheurer, PhD. Bénédicte Clement took the photographs and approved permission for use.

Day 2 continued with presentations from the ABTA Junior Investigator Award recipients, Joshua D. Strauss (US), from Baylor College of Medicine (Houston, Texas, USA) (Figure 1) and Raoull Hoogendijk (non-US), from Princess Máxima Center for Pediatric Oncology (Utecht, Netherlands) (Figure 2). These awards are given to researchers with meritorious abstracts who are in a trainee position (eg, student, postdoctoral research fellow, resident). Joshua gave a talk on **“*Novel Susceptibility Variants in Adult and Pediatric Ependymoma*”** and Raoull gave a talk on **“*Geographical Survival Comparison and Estimated Long-Term Survival Outcomes of Pediatric CNS Tumors from 31 European Countries – Results from the Population-Based EUROCARE Project*.”**

After the ABTA Junior Investigator Award presentations, day 2 continued with several abstract sessions (Table 1), a brainstorming session that allowed attendees to weigh in on topics for future BTEC meetings, and set important topics of research interest for BTEC members (https://bpb-us-w1.wpmucdn.com/sites.usc.edu/dist/9/231/files/2024/08/BTEC-2024-Mainz-Program-with-abstracts.pdf).

## Limitations and Challenges

While the BTEC annual meeting has been ongoing for over twenty years, there are a few critical challenges that we continue to address. There is a lack of attendance from some areas of the world at the annual meeting; this is an ongoing challenge due to financial constraints, work or travel permits, time commitments, and other institutional rules and regulations over meeting travel. To address this challenge, we have continued to offer hybrid meeting formats to all those investigators and trainees to attend even if they cannot travel. Furthermore, scientists from low- and middle-income countries (LMICs) are not as well represented. To address this challenge, in 2023, BTEC initiated a formal mentoring program to help provide training and research mentoring for promising junior scientists from LMICs who want to establish a career in brain tumor epidemiology research. Finally, the BTEC Board was assembled to be intentionally inclusive to international partners, alternating meeting locations every year to hopefully reduce travel burden. However, there have been continued challenges in our ability to ensure balanced participation. We continue to invest in overcoming these limitations to ensure our work reflects patients from all settings and backgrounds.

## Conclusions

The burden of brain tumors is particularly large in children, adolescents, and adults, especially amongst those diagnoses with the worse prognosis, such as high-grade gliomas. With improvements in treatment for several types of brain tumors, the proportion of this population that are long-term survivors is increasing. These survivors are at increasing risk of somatic late effects and other adverse outcomes as a result of their intense treatment regimens. Lastly, the experience and needs of survivors are increasingly relevant and need to be evaluated through expanded clinical care and research.

The BTEC 2024 annual meeting provided a forum for the focus on brain tumor survivorship across the age spectrum, in addition to our usual topics, including improvements in predicting brain tumor risk and survival. We will reconvene in 2025 for the annual meeting, “The Epidemiology of Brain Metastasis,” hosted by Quinn Ostrum at Duke University School of Medicine (Durham, North Carolina).
